# Transcriptome analysis reveals differences in mechanisms regulating cessation of luteal function in pregnant and non-pregnant dogs

**DOI:** 10.1186/s12864-017-4084-9

**Published:** 2017-09-27

**Authors:** Sophie Zatta, Hubert Rehrauer, Aykut Gram, Alois Boos, Mariusz Pawel Kowalewski

**Affiliations:** 1 Institute of Veterinary Anatomy, Vetsuisse Faculty, University of Zurich, Winterthurerstrasse 260, CH-8057 Zurich, Switzerland; 2 Functional Genomics Center Zurich, ETH Zurich/University of Zurich, Winterthurerstrasse 190, CH-8057 Zurich, Switzerland

**Keywords:** Dog (*Canis familiaris*), CL, Luteal regression, Prepartum luteolysis, Induced abortion

## Abstract

**Background:**

In the domestic dog, corpora lutea (CL) are the only source of progesterone (P4), both in pregnant and non-pregnant cycles because there is no placental steroidogenesis. The absence of an endogenous luteolysin in absence of pregnancy results in long-lasting physiological pseudopregnancy, strongly contrasting with the acute luteolysis observed prepartum. The underlying biological mechanisms and the involvement of P4 signalling remain, however, not fully understood. Therefore, here, next-generation sequencing (RNA-Seq) was performed on CL from the late luteal phase and compared with normally luteolyzing CL collected at the prepartum P4 decrease.

**Results:**

The contrast “luteal regression over luteolysis” yielded 1595 differentially expressed genes (DEG). The CL in late luteal regression were predominantly associated with functional terms linked to extracellular matrix (*p* = 5.52e-05). Other terms related to transcriptional activity (*p* = 2.45e-04), and steroid hormone signalling (*p* = 2.29e-04), which were more highly represented in late regression than during luteolysis. The prepartum luteolysis was associated with immune inflammatory responses (*p* = 2.87e-14), including acute-phase reaction (*p* = 4.10e-06). Immune system-related events were also more highly represented in CL derived from normal luteolysis (*p* = 7.02e-04), compared with those from dogs in which luteolysis was induced with an antigestagen (1480 DEG in total). Additionally, the withdrawal of P4 at mid-gestation resulted in 92 DEG; over-represented terms enriched in antigestagen-treated dogs were related to the inflammatory response (*p* = 0.005) or response to IL1 (*p* = 7.29e-05). Terms related to proliferation, e.g., centrosome organization (*p* = 0.002) and steroid metabolic processes (*p* = 0.001), prevailed at mid-gestation. Thereby, our results revealed the nature of luteotropic effects of P4 within canine CL. It appears that, even though they result in diminished steroidogenic output, the effect of antigestagens is more related to the withdrawal of P4 support than to the PGF2alpha-related inflammatory reaction observed at physiological parturition.

**Conclusions:**

We report the differential gene expression associated with maintenance and cessation of luteal function in pregnant and non-pregnant dogs. Based on the differentially expressed genes, we indicate functional pathways and gene networks that are potentially involved in the underlying endocrine and molecular mechanisms. This study establishes future research directions that may be helpful in understanding some of the clinical conditions, such as luteal insufficiency, associated with negative pregnancy outcome in dogs.

**Electronic supplementary material:**

The online version of this article (10.1186/s12864-017-4084-9) contains supplementary material, which is available to authorized users.

## Background

The domestic dog is classified as a mono-estric, polytocous and aseasonal breeder, with an obligatory quiescence phase called anestrus [1, 2]. Although canine reproduction has already attracted some attention, still many of the species-specific peculiarities remain not fully understood. Unlike in livestock, the dog lacks placental steroidogenesis [3]. Thus, luteal P4 is the only major source of circulating levels of this hormone, both in non-pregnant cycles and during pregnancy. The lifespan of corpora lutea (CL) in non-pregnant bitches often outlasts the luteal duration in pregnant animals [4]. The luteal phase and P4 levels are almost identical in both situations until around day 60, i.e., prior to parturition when a steep decline of serum P4 is observed in pregnant dogs, indicating prepartum luteolysis [5, 6]. In contrast, in the non-pregnant dog a slow decline of P4 is observed, until basal levels of P4 reach <1 ng/ml, indicating the onset of anestrus [4, 7]. Moreover, at least in non-pregnant dogs, the function of CL is independent of a uterine luteolysin (PGF2alpha), and remains unaffected by hysterectomy [8]. Also, a luteolytic function of intraluteally produced prostaglandins can be ruled out [9]. In pregnant dogs, the prepartum decline of P4 is concomitant with increased circulating levels of PGF2alpha, predominantly of fetal placental origin, indicating its role during parturition and/or luteolysis [3, 5, 6, 10, 11].

During the early luteal phase, canine CL exhibit decreased sensitivity to gonadotropic support [12, 13] and luteal PGE2 seems to be a potent luteotropic factor [14–16]. The period of relative gonadotropin independence ends at around day 25 post ovulation (p.o.) [13] and prolactin (PRL) becomes the main luteotropic factor thereafter [17, 18]. However, despite the continuously increasing availability of PRL and luteinizing hormone (LH), during the second half of diestrus luteal regression sets in, associated with structural degeneration of CL and without any signs of strong apoptosis [2, 19]. Consequently, based on the evidence so far, regression of the CL in the dog seems to be a passive degenerative process (reviewed in [1]). Only during prepartum luteolysis are the acute PGF2alpha surge and the concomitant steep fall of P4 associated with massive apoptotic activity within the CL [1]. Interestingly, there is no pregnancy- and/or parturition-related increase in estrogens in dogs [3]. The luteotropic role of P4 is substantiated by the fact that application of an antigestagen induces the utero-placental signaling cascade, unequivocally resulting in preterm luteolysis/abortion [2, 10, 20].

The role of the immune system in canine CL function is still not fully elucidated. Hoffmann and collaborators [21] reported the time-dependent presence of CD4- and CD8-positive immune cells along with cells staining positively for MHC II complex in the CL tissue of non-pregnant dogs. Additionally, expression of several interleukins (IL) and cytokines was confirmed at different stages of development in non-pregnant canine CL [22]. Recently, similar observations have been made with regards to canine CL of pregnancy [23], implying an active involvement of the immune system in the prepartum luteolytic cascade.

Nevertheless, regulatory mechanisms governing the canine CL lifespan, and especially differences in termination of CL function in pregnant vs. non-pregnant bitches, remain not fully understood. Therefore, aiming to obtain deeper insights into underlying endocrine regulatory mechanisms, herein, a next-generation deep RNA sequencing (RNA-Seq) was employed to investigate global gene expression during the maintenance and cessation of canine CL function. Luteal samples collected at prepartum luteolysis were compared with corresponding samples from non-pregnant dogs obtained during late CL regression (day 65 p.o.). The changes evoked by prepartum PGF2alpha release were assessed by comparing mid-gestation samples with those from active prepartum luteolysis. Additionally, in order to assess P4-dependent effects, samples were obtained from bitches in which pre-term luteolysis/abortion was induced at mid-term by the antigestagen aglepristone. The global gene expression in these samples was compared with natural prepartum luteolysis as well as with mid-pregnant dogs.

This is the first comprehensive, comparative transcriptome analysis of canine CL in pregnant and non-pregnant bitches during cessation of the CL life span, including a functional approach demonstrating antigestagen-mediated effects.

## Methods

### Animals, tissue sampling and preservation

All animal experiments and use of tissue samples were in accordance with animal welfare legislation and were approved by the respective authorities of the Justus-Liebig University, Giessen, Germany (permit no. II 25.3-19c20-15c GI 18/14 and VIG3-19c-20/15c GI 18,14) and the University of Ankara (permit no. 2006/06), Ankara, Turkey. All tissues were used in our previous experiments [24, 25]. Thus, corpora lutea (CL) from clinically healthy, cross-breed bitches (aged 2–8 years) were used representing the following experimental groups: (Group-1) mid-pregnancy (days 35–40 post ovulation, p.o.; *n* = 5); (Group-2) active prepartum luteolysis (*n* = 3); (Group-3) antigestagen-treated mid-gestation group (days 40–45 p.o.; *n* = 5); (Group-4) non-pregnant bitches at late CL regression (day 65 p.o.; *n* = 5).

In all dogs the time of ovulation was determined by measurements of plasma P4 (> 5 ng/ml) and by vaginal histology [26]. Pregnant dogs were mated 2 days after ovulation (Day 0), which in the dog is the time needed for completion of oocyte maturation within the oviduct. To determine active prepartum luteolysis (Group-2), P4 concentrations in peripheral blood plasma were measured at 6 h intervals beginning on day 58 of pregnancy; when P4 levels in 3 consecutive measurements fell below 2–3 ng/ml, the tissue material was collected. The respective P4 levels are presented in [10]. In Group 3, prepartum luteolysis/abortion was induced with the antigestagen aglepristone (Alizine(R), Virbac, Bad Oldesloe, Germany; 10 mg/Kg bw, 2×/24 h apart) and the tissues were collected 24 h after the second application.

All dogs underwent routine ovariohysterectomy. Immediately after surgery, the CL tissues were trimmed of surrounding ovarian tissue, washed with phosphate-buffered saline (PBS) and placed in RNAlater(R) (Ambion Biotechnologie GmbH, Wiesbaden, Germany) for 24 h at +4 °C, and then stored at −80 °C until use.

Tissues from all animals were used for RNA-Seq and TaqMan PCR experiments. Because of limited tissue material in Group-4, only three of five samples were used for RNA-Seq. Due to formal restrictions regarding collection of tissue material and experimental procedures, Group-2 (prepartum luteolysis) was comprised of only three replicates.

### RNA isolation and purification

Total RNA was extracted using TRIzol(R) Reagent following the manufacturer’s protocol (Invitrogen, Carlsbad, CA, USA). The RNA purity and quantity was measured with a NanoDrop 2000C(R) spectrophotometer (Thermo Fischer Scientific AG, Reinach, Switzerland). Further purification of RNA was performed by the RNeasy® Mini Kit (Qiagen GmbH, Hilden, Germany). RNA integrity was assessed with the Agilent 2200 TapeStation System. The RNA integrity numbers (RIN) ranged from 7.2 to 9.5.

### RNA-sequencing and data evaluation

#### Library preparation

The quality and quantity of isolated RNA were determined with a Qubit® (1.0) Fluorometer (Life Technologies, California, USA) and a Bioanalyzer 2100 (Agilent, Waldbronn, Germany). The TruSeq RNA Sample Prep Kit v2 (Illumina, Inc., California, USA) was used in the succeeding steps. Briefly, total RNA samples (100-1000 ng) were poly-A enriched and then reverse-transcribed into double-stranded cDNA. The cDNA samples were fragmented, end-repaired and polyadenylated before ligation of TruSeq adapters containing the index for multiplexing. Fragments containing TruSeq adapters on both ends were selectively enriched with PCR. The quality and quantity of enriched libraries were validated using the Qubit(R) (1.0) Fluorometer and the Caliper GX LabChip(R) GX (Caliper Life Sciences, Inc., USA). The product is a smear with an average fragment size of approximately 260 bp. The libraries were normalized to 10 nM in Tris-Cl 10 mM, pH 8.5 with 0.1% Tween 20.

#### Cluster generation and sequencing

The TruSeq PE Cluster Kit v4-cBot-HS or TruSeq SR Cluster Kit v4-cBot-HS (Illumina, Inc., California, USA) were used for cluster generation using 10 pM of pooled normalized libraries on the cBOT System. Sequencing was performed on the Illumina HiSeq 2500 single end 125 bp using the TruSeq SBS Kit v4-HS (Illumina, Inc.). The data described in this publication have been deposited in NCBI’s Gene Expression Omnibus and are accessible through GEO Series accession number GSE98657 (https://www.ncbi.nlm.nih.gov/geo/query/acc.cgi?token=azclmwquvjwftsl&acc=GSE98657).

#### Data analysis

For the quantitative assessment of gene expression, next generation sequencing (NGS, RNA-Seq) was performed. To align the large transcriptome RNA-Seq dataset, the Spliced Transcripts Alignment to Reference (STAR-aligner) software was used to perform RNA-Seq data read-alignment [27]. As for the reference genome, the Ensembl genome build CanFam3.1 was used (www.ensembl.org/Canis_familiaris/Info/Index). With the function *featureCounts* from the R package Rsubread, the gene expression values were calculated [28]. We considered a gene as detected if it had an average count of 10 reads in at least one group of replicates. Differential expression was assessed using the generalized linear model approach implemented in the Bioconductor package DESeq2 [29]. Specifically, we used a call to the function DESeq2, which: (a) provides an estimate of size factors to normalize for sequencing depth, (b) estimates the dispersion function for the expression counts, and finally (c) performs a fit with a negative binomial model that uses the experimental groups as a single factor. Significance of the differential expression was assessed using the Wald test for the coefficients of the fitted model. Details are described in the documentation of the Bioconductor package DESeq2 [29]. The *p*-value was adjusted to <0.01. Next, the “FDR”-method (False Discovery Rate; FDR 10%, i.e. adjusted *p*-value <0.1) was computed using the Benjamini-Hochberg algorithm and applied for correction of multiple testing. Differentially expressed genes (DEG) were identified for the selected contrasts, i.e., pairwise comparisons. Complete results are provided in Additional files 1, 2, 3 and 4. With these settings and the expected variability of results, in particular following antigestagen treatment, our approach was explorative allowing us to select potential DEG for further downstream analysis by qPCR. Accordingly, the expression of selected key target genes was validated by RT-PCR. Association of Gene Ontology (GO) categories with DEG was computed using the Bioconductor package *goseq* [30]. In order to detect significantly enriched biological pathways, the RNA-Seq data were further analyzed with the web-based software QIAGEN’s Ingenuity(R) Pathway Analysis (IPA(R), QIAGEN Redwood City, build version: 364,062 M, content version: 26,127,183 (release date: 2015-11-30)). Additionally, Partek(R) Genomics Suite(R) (version 6.6 Copyright(C); 2015 Partek Inc., St. Louis, MO, USA), a next generation sequencing, microarray and qPCR data analysis software, and Enrichr, an integrative web-based and mobile software application [31], were applied to support and corroborate the results. With the open source bioinformatics software platform Cytoscape v3.0.0 [32] application ClueGO v2.2.3 [33], analysis and visualization of possible differentially enriched functional biological networks were performed for the following contrasts (i.e., pairwise group comparisons): “luteal regression over luteolysis”, and “luteolysis over antigestagen”. As input for IPA and Cytoscape, all DEG with *p* < 0.01, FDR < 0.1 for the respective contrasts were used (Table 2, respective Additional files 1, 2, 3 and 4). The Venn diagrams show the overlap of DEG when applying an additional fold-change threshold of two-fold up- and down-regulation.

### Expression of selected target genes by semi-quantitative real time (TaqMan) polymerase chain reaction (PCR)

Total RNA from all samples was used for semi-quantitative RT-PCR. With each sample, 10 ng of RNA was used. DNase treatment was performed following the instructions of the manufacturer (Promega, Dübendorf, Switzerland) with RQ1 RNase-free DNase. Reverse transcription (RT) was carried out with the supplier’s protocol for the High Capacity cDNA Reverse Transcription Kit including RNase Inhibitor (Applied Biosystems from Thermo Fisher Scientific, Foster City, CA, USA). The quantity of cDNA was then increased by amplifying it according to the protocol for the TaqMan® PreAmp Master Mix Kit (Applied Biosystems).

TaqMan PCR was performed as described previously [10, 34, 35]. Gene-specific primers and 6-carboxyfluorescein (6-FAM) and 6-carboxytetramethyl-rhodamine (TAMRA) labeled probes were designed using PrimerExpress software version 2.0 (Applied Biosystems) ordered from Microsynth, Balgach, Switzerland. The efficiency of self-designed expression assays was validated as previously described [35] ensuring approximately 100%. The predesigned TaqMan systems were ordered from Applied Biosystems by Thermo Fisher Scientific. For an overview of the primers, TaqMan probes and predesigned TaqMan systems see Table 1. Reactions were run in an automated fluorometer ABI PRISM^®^ 7500 Sequence Detection System (Applied Biosystems). As a control, experiments were run in the absence of the enzyme during the reverse transcription step (the so-called minus-RT control). Relative quantification was done using the comparative CT method (ΔΔ CT method) [34, 35]. GAPDH, B-ACTIN and Cyclophilin A (PPIA) acted as reference genes for normalization of target gene expression [34, 35]. The sample with the lowest expression of the target gene served as the calibrator. Relative gene expression (RGE) is presented. The threshold cycle (CT) value constitutes the PCR cycle number at which the reported fluorescence exceeds the base line above the background fluorescence. Due to the uneven distribution of Real Time data, logarithmic transformation was performed and the results are presented as geometric means (Xg) +/− geometric standard deviation (SD). An unpaired, two-tailed Student’s t-test was applied to show pairwise contrasts of gene expression between groups. GraphPad3 (GraphPad Software Inc., San Diego, CA, USA) was used; *p* < 0.05 was considered as significant.

**Table 1:** List of primers used for Real Time (TaqMan) PCR

Primer	Accession number	Primer sequence	Product length (bp)
SAA-for SAA-rev SAA-TaqMan Probe	NM_001313872.1	5′-TGG GAC ATG TTG AGA GCC TAC TC-3′ 5′-CCT CTG TGC AGC GTC ATA GTT C-3′ 5′-TTC AGA CAA ATA CTT CCA TGC CCG GG-3’	114
CXCL8-for CXCL8-rev CXCL8-TaqMan Probe	NM_001003200.1	5’-CCA CAC CTT TCC ATC CCA AA-3′ 5′-CCA GGC ACA CCT CAT TTC CA-3′ 5′-CTG AGA GTG ATT GAC AGT GGC CCA CAT TGT-3’	114
IL1b-for IL1b-rev IL1b-TaqMan Probe	NM_001037971.1	5’-TGC CAA GAC CTG AAC CAC AGT-3′ 5′-CTG ACA CGA AAT GCC TCA GAC T-3′ 5′-CAT CCA GTT GCA AGT CTC CCA CCA GC-3’	97
GAPDH-for GAPDH-rev GAPDH-TaqMan Probe	NM_001003142.2	5’-GCT GCC AAA TAT GAC GAC ATC A-3′ 5′-GTA GCC CAG GAT GCC TTT GAG-3′ 5′-TCC CTC CGA TGC CTG CTT CAC TAC CTT-3’	75
MHCII-for MHCII-rev MHCII-TaqMan Probe	NM_001011723.1	5’-GGA GAG CCC AAC ATC CTC ATC-3′ 5′-GGT GAC AGG GTT TCC ATT TCG-3′ 5′-TCG ACA AGT TCT CCC CAC C-3’	90
RXFP1-for RXFP1-rev RXFP1-TaqMan Probe	XM_014119744.1	5’-GGC ACC AAT GGA GTG TGT TTC-3′ 5′-TGC CGC CAA GTT AAC ACC AA-3′ 5′-TAC TGG AGC CCA GAT TTA TTC GGT GGC-3’	102
GATA 4	NM_001048112.1	Applied Biosystems, prod. nr.: Cf02736086_m1	104
JUN	XM_860558.1	Applied Biosystems, prod. nr.: Cf02696722_g1	90
CCL13	NM_001003966.1	Applied Biosystems, prod. nr.: Cf02622470_mH	117
CCL3	NM_001005251.1	Applied Biosystems, prod. nr.: Cf02671956_m1	131
YY1	XM_849421.1	Applied Biosystems, prod. nr.: Cf02637858_m1	81
ECM2	XM_533562.2	Applied Biosystems, prod. nr.: Cf02641132_m1	74
GATA6	XM_547642.2	Applied Biosystems, prod. nr.: Cf02654912_m1	71
CCNA2	XM_540965.2	Applied Biosystems, prod. nr.: Cf02648449_g1	77
B-ACTIN	NM_001003349.1	Applied Biosystems, prod. nr.: Cf03034055_u1	121
PPIA	XM_843327.1	Applied Biosystems, prod. nr.: Cf03986523_gH	92

## Results

### Analysis of RNA-Seq data

Pairwise comparisons (contrasts) of NGS data were performed for selected experimental groups. The normalized read count data were produced with the Bioconductor package DEseq2 [29]. A summary of the results for all contrasts investigated in the study is presented in Table 2. The complete result tables are available as Additional files 1, 2, 3 and 4.

**Table 2:** Summary of the RNA-Seq results for all contrasts investigated in the study, feature counts including DEG (differentially expressed genes) numbers, are presented

Analysis	Luteal regression over luteolysis	Mid-gestation over luteolysis	Mid-gestation over antigestagen	Luteolysis over antigestagen
Genes Total (*p*-value <0.01, FDR <0.1)	1595	1745	92	1480
Genes UP	541	690	38	793
Genes Down	1054	1055	54	687
Number of features	23,630	23,630	23,630	23,630
Number of features with counts above threshold	14,883	14,469	14,186	14,280
Linear signal threshold	10	10	10	10

Contrast: “luteal regression over luteolysis”.

The number of features with counts above threshold of 10 reads per gene was 14,883. We call differentially expressed genes (DEG) by applying a *p*-value threshold of 0.01 and adjusted *p*-value (FDR) of 0.1 and found 1595 significant DEG: 1054 genes show lower and 541 show higher expression in the luteal regression group compared to the luteolysis group.

Contrast: “mid-gestation over luteolysis”.

In this contrast, the number of features with counts above the threshold 10 reads per gene was 14,469. Of the 1745 DEG (*p*-value < 0.01, and adjusted *p*-value, FDR < 0.1) found in this contrast, 690 were more highly expressed and 1055 were lower at mid-gestation compared to the luteolysis group.

Contrast: “mid-gestation over antigestagen”.

The number of features with counts above the threshold of 10 reads per gene was 14,186. This contrast generated the highest variability in gene expression levels and the least number of genes that were differentially expressed. Of the 429 genes initially selected based on the *p* < 0.01 criterion, only 92 DEG passed the FDR < 0.1 correction and were therefore used for all downstream analyses; 38 genes were more and 54 genes were less expressed in mid-gestation compared to the antigestagen group. Although expected, the high variability of gene expression limits the interpretability of results.

Contrast: “luteolysis over antigestagen”.

For this contrast, the number of features with counts above the threshold was 14,280. From the 1480 DEG (*p* < 0.01, FDR < 0.1) identified in this contrast, 687 were more and 793 were less expressed in the antigestagen group in contrast to the luteolysis group.

#### Functional annotations and pairwise comparisons

**Fig. 1:**
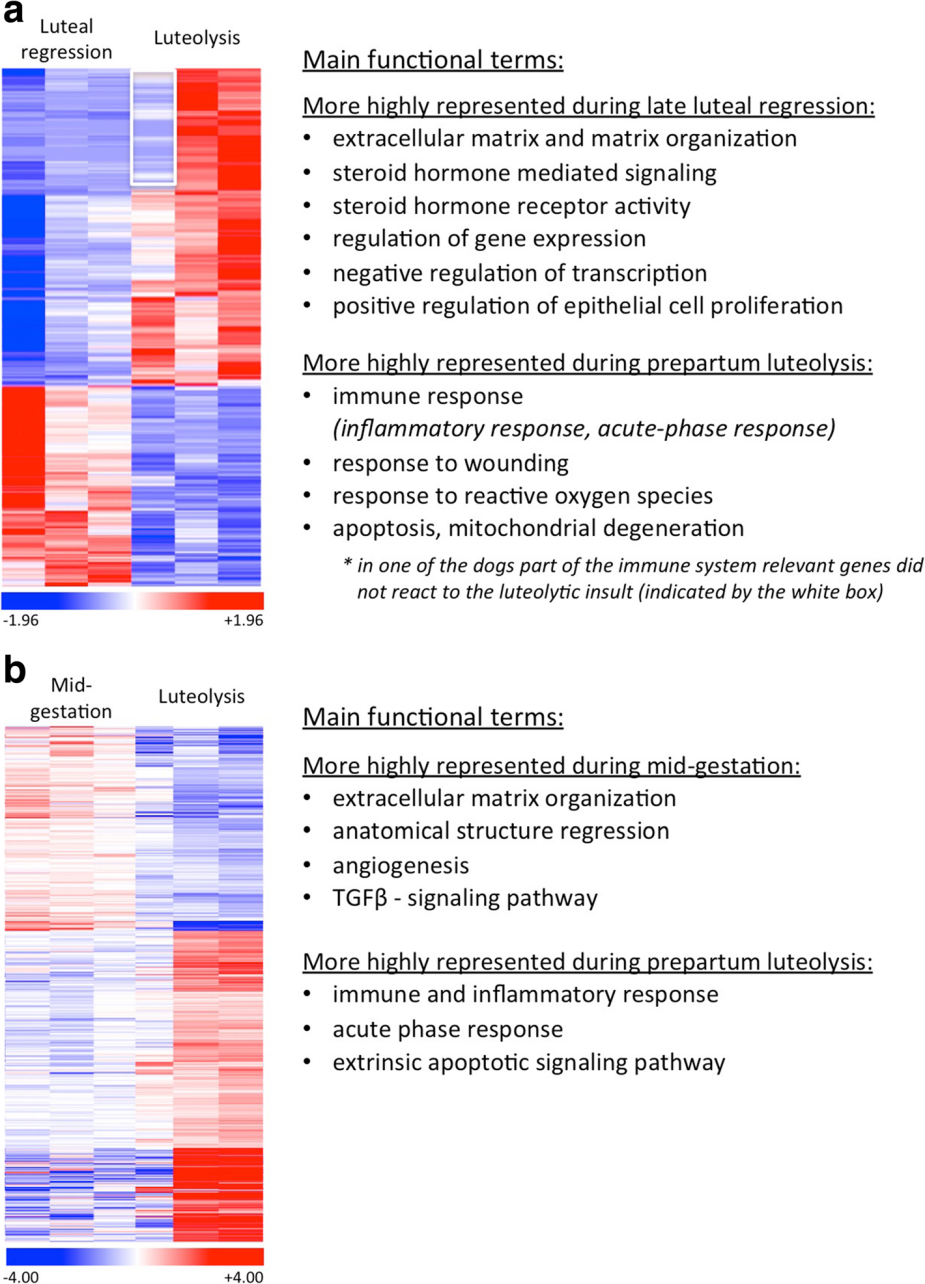
Representative heatmaps showing the RNA-Seq analysis of differentially expressed genes (DEG) of two contrasts: **a** “luteal regression over luteolysis”, and **b** “mid-gestation over luteolysis”. For each gene the red color indicates high expression relative to the average expression of the gene, while the blue color indicates low expression. **a** 1595 DEG were detected for the contrast “luteal regression over luteolysis”, 1054 genes were less and 541 were more highly expressed in luteal regression (*p* < 0.01, FDR < 0.1), **b** 1745 DEG were found in the contrast “mid-gestation over luteolysis”, 1055 were less and 690 were more expressed in mid-gestation (*P* < 0.01, FDR < 0.1). The main functional terms overrepresented in each of the groups are listed (details, including statistics are presented in the text). The entire list of DEG identified genes is provided as Additional files 1 and 2

**Fig. 2:**
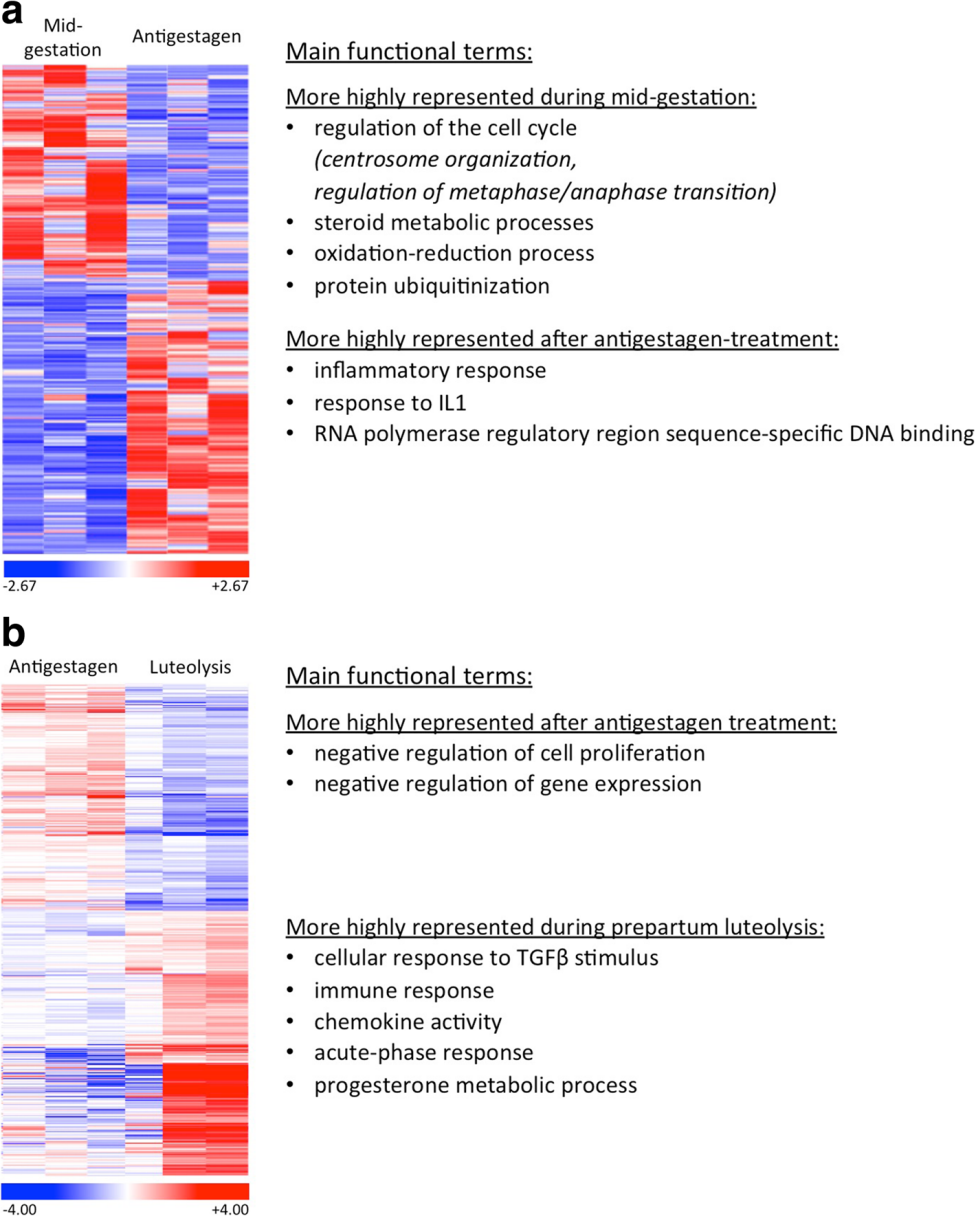
Representative heatmaps showing the RNA-Seq analysis of differentially expressed genes (DEG) of two contrasts: **a** “mid-gestation over antigestagen”, and **b** “luteolysis over antigestagen”, are presented. For each gene the red color indicates high expression relative to the average expression of the gene, while the blue color indicates low expression. **a** 492 genes were found for the contrast “mid-gestation over antigestagen” based on the *p* < 0.01 threshold (shown). Of these, 92 DEG passed the FDR < 0.1 selection (adjusted *p*-value), of which 38 genes were more and 54 genes were less expressed at mid-gestation. These genes were used for further downstream analyses. **b** 1480 DEG were identified in the contrast “luteolysis over antigestagen”, 793 were more and 687 were less expressed in the antigestagen group compared to the luteolysis group (*p* < 0.01, FDR < 0.1). The main functional terms overrepresented in each of the groups are listed (details, including statistics are presented in the text). The entire list of DEG identified genes is provided as Additional files 3 and 4

We identified the Gene Ontology (GO) terms that are enriched with differentially expressed genes in the three subgroups: biological process (BP), cellular compartment (CC) and molecular function (MF). Figs. 1 and 2 show heatmaps of the differentially expressed genes together with associated main functional terms. Lists of representative genes involved in particular GO terms in each contrast are presented in Additional file 5.

“Luteal regression over luteolysis”:

GO terms having highly significant association with genes upregulated in the luteal regression group are: extracellular matrix (*p* = 5.52e-05) and matrix organization (*p* = 0.001), steroid hormone mediated signaling (*p* = 2.29e-04) and steroid hormone receptor activity (*p* = 1.63e-04), regulation of gene expression (*p* = 7.40e-04), negative regulation of transcription from RNA polymerase II promoter (*p* = 5.02e-05), and regulation of transcription, DNA-templated (*p* = 2.45e-04), as well as positive regulation of epithelial cell proliferation (*p* = 3.4e-03).

Terms associated with genes that were more highly expressed in prepartum luteolysis were: immune response (*p* = 1.35e-14) (e.g., leucocyte migration, chemotaxis) and inflammatory response (*p* = 2.87e-07), regulation of response to wounding (*p* = 1.41e-05), response to reactive oxygen species (*p* = 0.002), acute-phase response (*p* = 4.10e-06), positive regulation of angiogenesis (*p* = 0.003), and apoptotic mitochondrial changes (*p* = 0.02).

“Mid-gestation over luteolysis”:

More represented during mid-gestation were terms relating to extracellular matrix organization (*p* = 0.002), anatomical structure regression (*p* = 0.01), angiogenesis (*p* = 0.03) and TGFβ signaling pathway (*p* = 4.47e-06). In contrast, represented more highly during prepartum luteolysis were: immune (*p* = 5.34e-08) and inflammatory response (*p* = 1.23e-04) (e.g., regulation of lymphocyte migration, positive regulation of defense response, chemokine-mediated signaling, cytokine activity, leukocyte cell-cell adhesion), acute-phase response (*p* = 0.003) and extrinsic apoptotic signaling pathway (*p* = 0.02).

“Mid-gestation over antigestagen”:

In this contrast, functional terms enriched in mid-gestation referred in general to: centrosome organization (*p* = 0.002), regulation of mitotic metaphase/anaphase transition (*p* = 7.34e-05), the oxidation-reduction process (*p* = 0.002), steroid metabolic processes (*p* = 1.38e-03) or protein ubiquitination (*p* = 0.006). The antigestagen-treated group included enriched GO terms such as: inflammatory response (*p* = 0.005), response to IL1 (*p* = 7.29e-05) and RNA polymerase regulatory region sequence-specific DNA binding (*p* = 0.004).

“Luteolysis over antigestagen”:

This contrast revealed the presence of the following main terms represented more during active prepartum luteolysis: cellular response to TGFβ stimulus (*p* = 3.00e-04), functional terms related to immune response (*p* = 7.02e-04), and chemokine activity (*p* = 4.68e-05), acute-phase response (*p* = 0.009) and progesterone metabolic process (*p* = 0.01). The terms that were represented more in the antigestagen-treated group (i.e., during induced abortion/luteolysis) related to negative regulation of cell proliferation (*p* = 2.04e-04) and negative regulation of gene expression (*p* = 0.002).

#### Venn diagrams

**Fig. 3:**
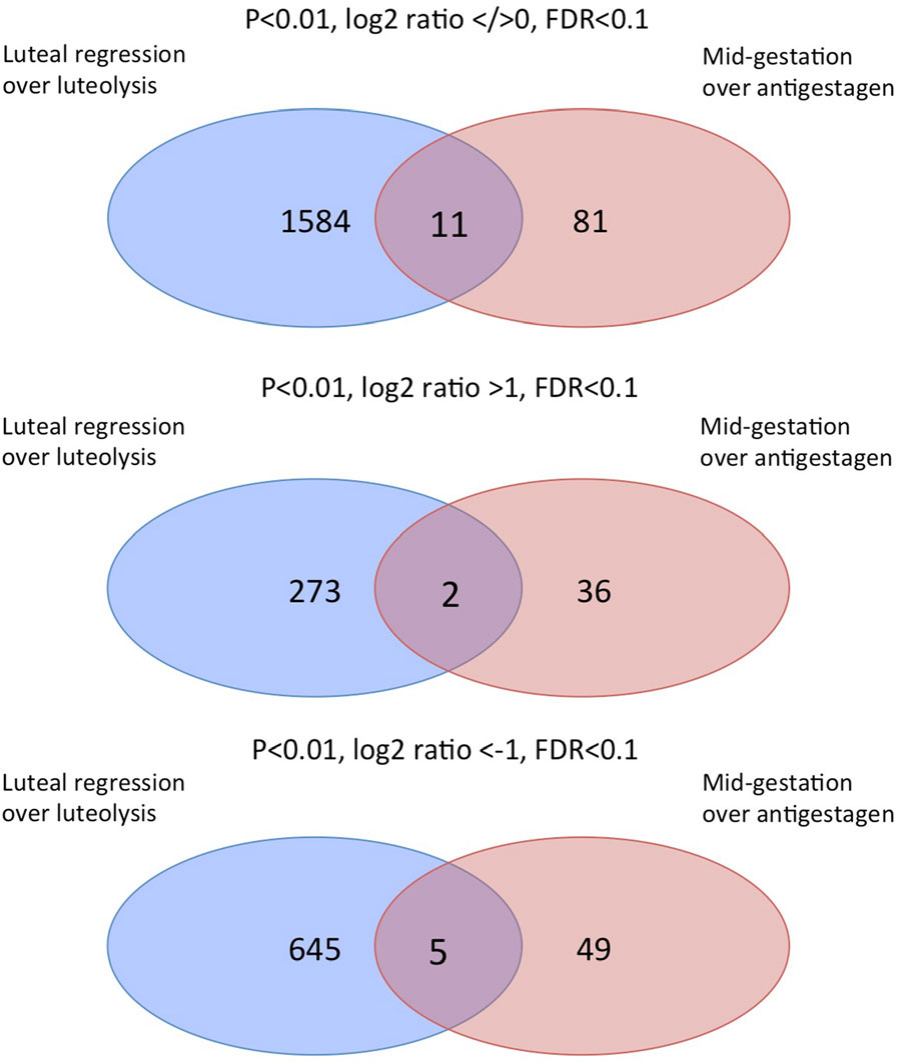
Venn diagram showing the intersection of differentially expressed genes (DEG) in the contrast “luteal regression over luteolysis” compared with the contrast “mid-gestation over antigestagen”. Lists of DEG (*p* < 0.01, FDR < 0.1) presented in Additional files 1 and 3 were used. Three analyses are presented: with up- and down-regulated genes (regardless of log2 ratio), or with upregulated genes (log2 > 1), or with downregulated genes (log2 < −1). When full sets of genes were used (up and down regulated), 11 genes were found overlapping in both contrasts, 1584 genes were found only in the contrast “luteal regression over luteolysis” and 81 genes were found in “mid-gestation over antigestagen”. Adding log2 ratio > 1 to the threshold, 2 over-represented genes overlapped in both contrasts, and for log2 ratio < −1, 5 downregulated genes were found

**Fig. 4:**
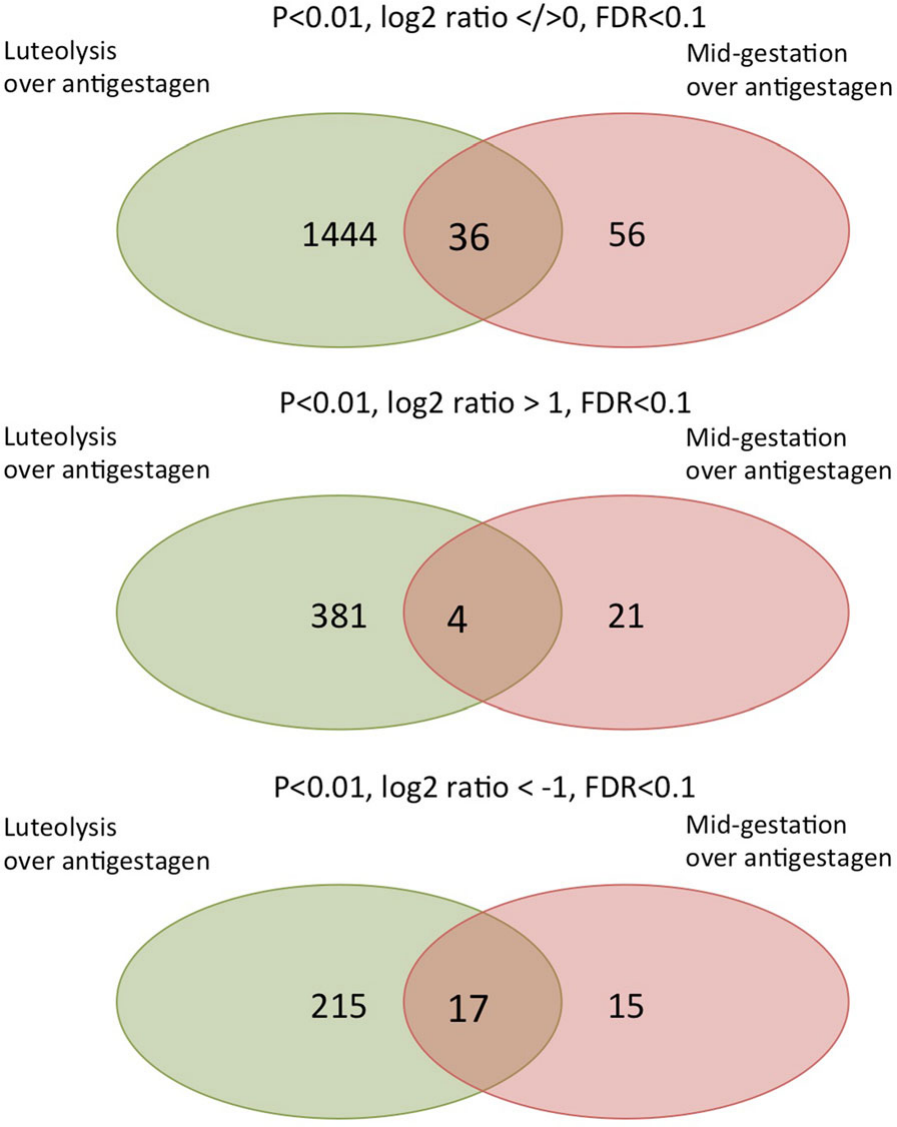
Venn diagram showing the intersection of differentially expressed genes (DEG) in the contrast “luteolysis over antigestagen” compared with the contrast “mid-gestation over antigestagen”. Lists of DEG (*p* < 0.01, FDR < 0.1) presented in Additional files 3 and 4 were used. Three analyses are presented: with up- and down-regulated genes (regardless of log2 ratio), or with upregulated genes (log2 > 1), or with downregulated genes (log2 < −1). When full sets of genes were used (up and down regulated), 36 genes were identified in both contrasts, 1444 genes were found in “luteolysis over antigestagen” only, and 56 genes in “mid-gestation over antigestagen”. Setting the log2 ratio > 1, 4 genes commonly over-represented in both comparisons were found, and with log2 ratio < −1, 17 commonly downregulated genes were detected

We illustrated the intersections among genes differentially expressed between the following selected contrasts: “luteal regression over luteolysis” vs. “mid-gestation over antigestagen” (Fig. 3), and “luteolysis over antigestagen” vs. “mid-gestation over antigestagen” (Fig. 4). All genes derived from the respective lists of DEG were used as input (Additional files 1, 3 and 4). For the first comparison, 11 genes fulfilling the criteria (DEG *p* < 0.01, FDR < 0.1 regardless of log2 ratio) were found overlapping in both contrasts, 1584 genes were found solely in the contrast “luteal regression over luteolysis” and 81 genes in the contrast “mid-gestation over antigestagen” (Fig. 3). When genes upregulated in each gene set were taken into consideration, additionally using the criteria log2 ratio > 1, 2 genes (CYP17A1, SULT1E1) that were commonly overrepresented overlapped in both contrasts, meaning that the same two upregulated genes can be found in both contrasts. On the other hand, setting the log2 ratio < −1, 5 commonly downregulated DEG were identified in both contrasts (ZSWIM4, C5AR2, TTC9, ADM5, C10orf10). Regarding the Venn diagrams, which visualize comparison of the two contrasts “luteolysis over antigestagen” vs. “mid-gestation over antigestagen” (Fig. 4), 36 DEG (*p* < 0.01, FDR < 0.1 regardless of log2 ratio) occurred in both contrasts. For log2 ratio > 1, 4 upregulated genes (CYP17A1, HJURP, AOC1, NT5E) were found in both contrasts, and with log2 ratio < −1, 17 commonly downregulated genes were identified (e.g., FZD5, TMEM65, CSRNP3, RASEF, TRPC4, CLCN1, C10orf10, NFIL3, KIF5C, ELL2). Detailed lists of overlapping genes are presented in Additional file 6.

#### Cytoscape analysis of functional networks

**Fig. 5:**
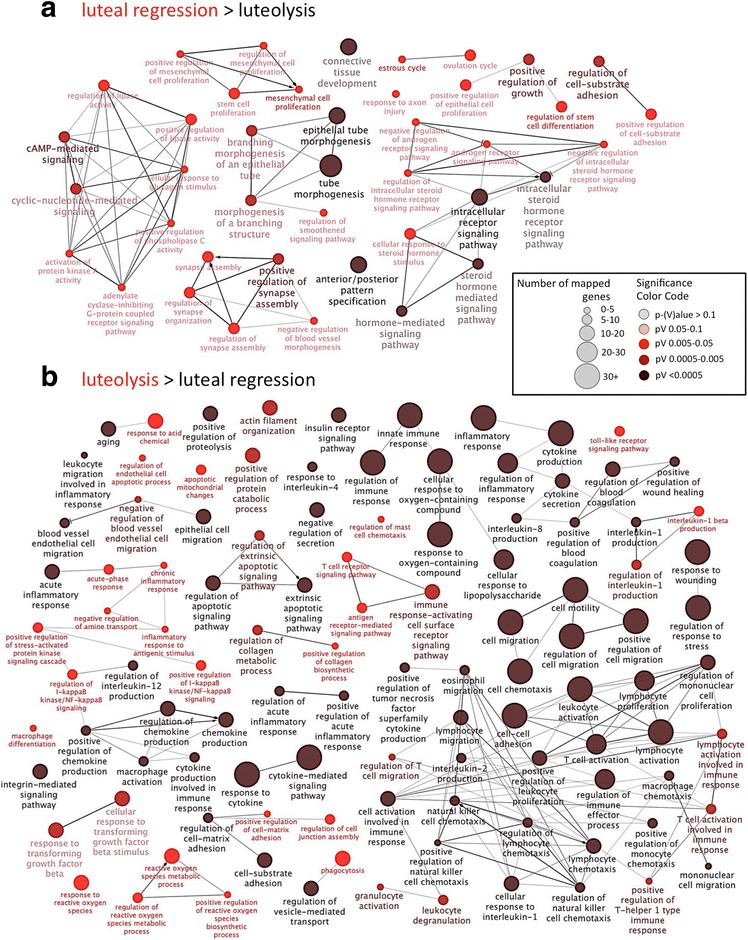
Cytoscape analysis of GO for the contrast “luteal regression over luteolysis” is presented. The functional terms overrepresented in each of the groups are shown. As input differentially expressed genes (DEG) were used (threshold was set at *p* < 0.01, FDR < 0.1). The redundant and non-informative terms were removed, and the resulting network was manually rearranged. For each network the size of the node implies the number of genes, while the color intensity denotes the level of enrichment (see legend to illustration). Functional networks, which were more highly represented in the luteal regression group (**a**), refer predominantly to matrix remodeling, to the steroid hormone signaling pathway and to cAMP-mediated signaling. Networks more highly represented during prepartum luteolysis (**b**) were associated to immune system, inflammatory response and regulation of apoptotic signaling

**Fig. 6:**
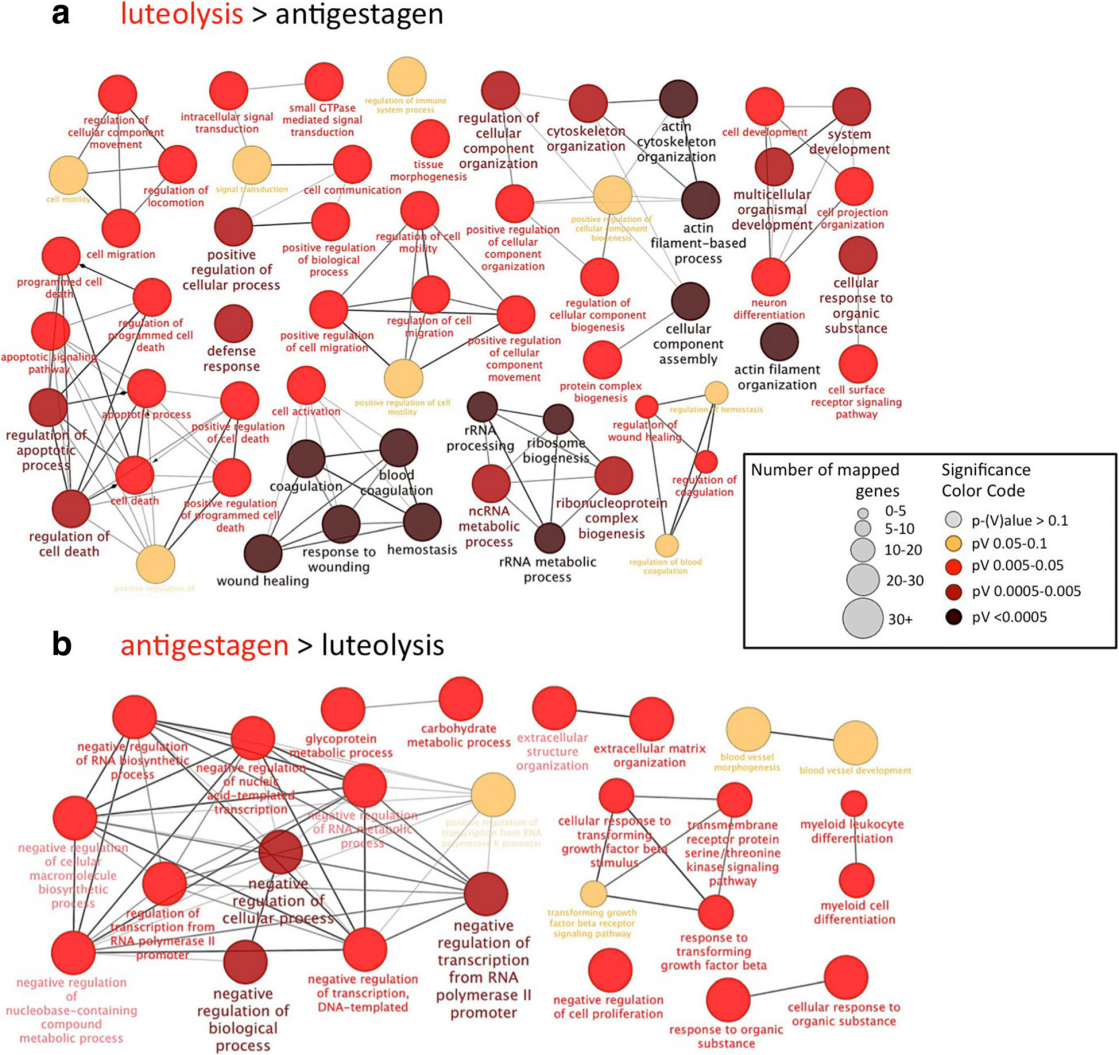
Cytoscape analysis of GO for the contrast “luteolysis over antigestagen” is presented. The functional terms overrepresented in each of the groups are shown. As input differentially expressed genes (DEG) were used (threshold was set at *p* < 0.01, FDR < 0.1). The redundant and non-informative terms were removed, and the resulting network was manually rearranged. For each network the size of the node implies the number of genes, while the color intensity denotes the level of enrichment (see legend to illustration). **a** The more highly represented functional networks for the prepartum luteolysis group include, e.g., response to wounding, defense response, positive regulation of cellular process, cytoskeleton organization and apoptotic signaling, as well as cell death. **b** The functional networks more represented in the antigestagen-treated group refer, e.g., to the negative regulation of transcription and networks relating to response to TGFβ

We used Cytoscape to visualize the correlation of functional networks associated with differentially expressed genes. We present the results for two contrasts: “luteal regression over luteolysis” and “luteolysis over antigestagen” in order to determine pathways potentially involved in normal and induced parturition (Figs. 5 and 6). As input, all DEG found for the respective contrasts were used (Table 2, Additional files 1 and 4; threshold *p* < 0.01, FDR < 0.1). Lists of representative genes involved in particular networks are presented in Additional file 5.

As for the contrast “luteal regression over luteolysis” (Fig. 5), among the predominant functional networks represented more in the luteal regression group were those networks referring to matrix remodeling (up to 10 representative genes are shown in alphabetical order), e.g., epithelial tube morphogenesis, connective tissue development, steroid hormone signaling pathways and cellular responses to steroidogenic stimulus, as well as cAMP-mediated signaling. On the other hand, the vast majority of more highly represented functional networks detected during prepartum luteolysis were those related to the immune system, e.g., regulation of the immune response, response to cytokines, leukocyte activation, lymphocyte activation, inflammatory response, and regulation of apoptotic signaling pathways.

Regarding the next contrast “antigestagen over luteolysis” (Fig. 6), the major functional network overrepresented in the antigestagen-treated group related to the negative regulation of transcription, e.g., negative regulation of transcription from RNA polymerase II promoter, negative regulation of cell proliferation, and networks relating to the response to TGFβ. The functional networks predominantly detected during active prepartum luteolysis in this contrast included the response to wounding, defense response and positive regulation of cellular processes. Other strongly represented networks include cytoskeleton organization and actin cytoskeleton organization, as well as functional networks relating to apoptotic signaling and regulation of cell death.

#### Ingenuity pathway analysis (IPA)

Lists of significant genes (DEG *p* < 0.01, FDR < 0.1 ) of interest were uploaded into IPA software. Lists of representative pathways, genes and top upstream regulators are presented in Additional file 5. For the contrast “luteal regression over luteolysis”, among the top overrepresented canonical pathways induced during prepartum luteolysis were: granulocyte adhesion and diapedesis (*p* = 2.4e-11), agranulocyte adhesion and diapedesis (*p* = 9.13e-09) and integrin signaling (*p* = 1.12e-08). The top upstream regulators, which were all defined as inhibited during luteal regression, i.e., predicted to be induced during luteolysis were: lipopolysaccharide (*p* = 5.45e-34), TNF (*p* = 3.12e-29), TGFB1 (*p* = 9.68e-29), IFNG (*p* = 2.86e-26) and β-estradiol (*p* = 2.66e-23).

Regarding the contrast “mid-gestation over luteolysis”, one of the predominant top canonical pathways represented more in luteolysis was NFκB signaling (*p* = 2.09e-09), and the top upstream inhibited regulators for mid-gestation, i.e., induced during luteolysis, were: TGFB1 (*p* = 6.86e-39), TNF (*p* = 5.62e-31), β-estradiol (*p* = 2.97e-28), lipopolysaccharide (*p* = 4.20e-26) and IFNG (*p* = 2.44e-23).

Due to the low number of input genes, no enriched canonical pathways were identified in the contrast “mid-gestation over antigestagen”. The top upstream regulators included, however, e.g., NUPR1 (*p* = 6.80e-06) or SOD1 (*p* = 1.96e-04).

The top canonical pathways for the last contrast “luteolysis over antigestagen” included: signaling by Rho family GTPases (*p* = 1.17e-16) and integrin signaling (*p* = 4.06e-06), which were more highly represented in normal prepartum luteolysis. The top upstream regulators which were activated during luteolysis were TGFB1 (*p* = 4.33e-32) and TNF (*p* = 1.64e-18).

### Expression of genes by semi-quantitative (TaqMan) RT-PCR

**Fig. 7:**
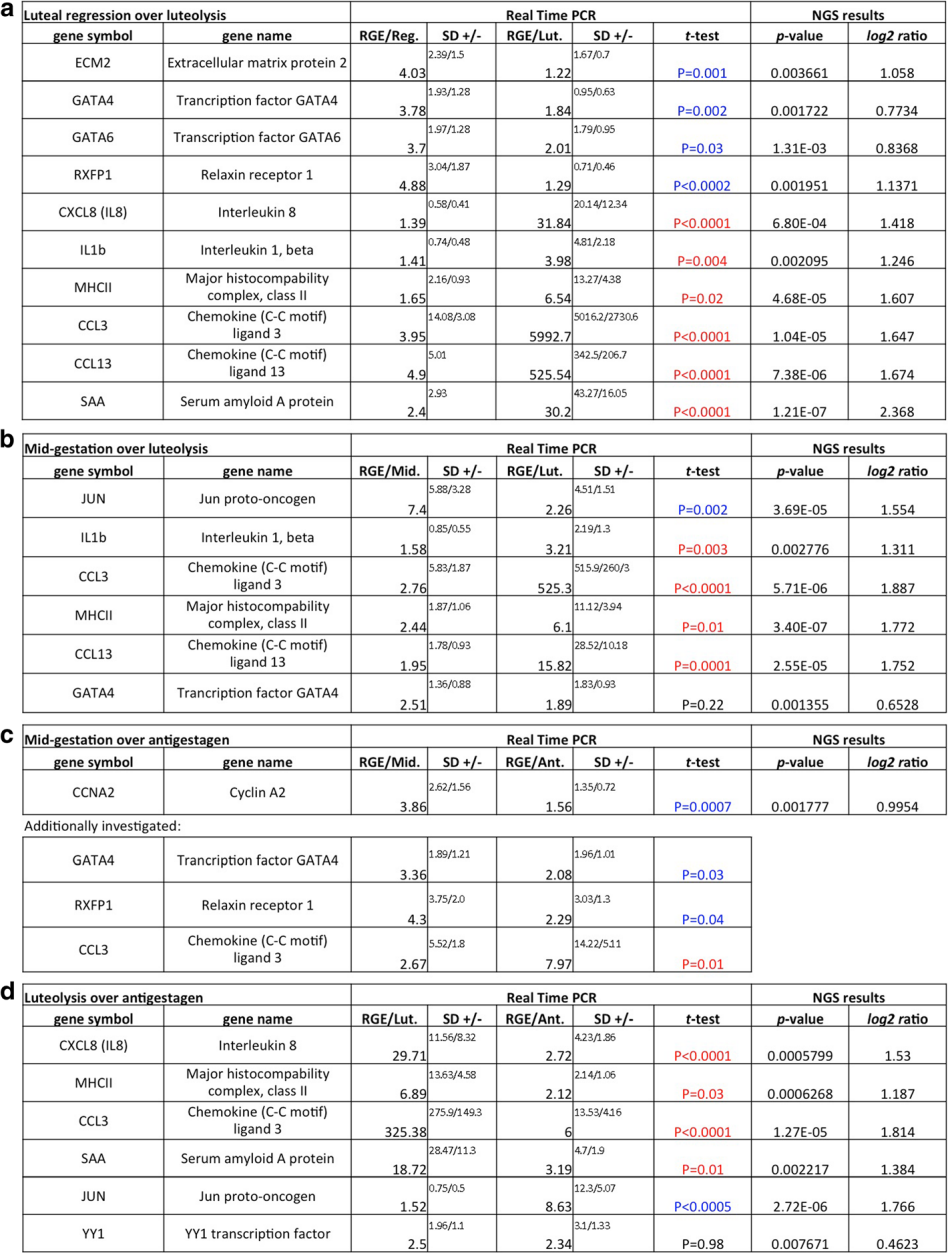
Expression of selected target genes for each of the investigated contrasts, as determined by Real Time (TaqMan) PCR. **a** “luteal regression over luteolysis” (t-test: blue = luteal regression > luteolysis; red = luteolysis > luteal regression), **b** “mid-gestation over luteolysis” (t-test: blue = mid-gestation > luteolysis; red = luteolysis > mid-gestation; black = unchanged), **c** “mid-gestation over antigestagen-treatment” (t-test: blue = mid-gestation > antigestagen; red = antigestagen > mid-gestation), and **d** “antigestagen-treatment over luteolysis” (t-test: red = luteolysis > antigestagen; blue = antigestagen > luteolysis; black = unchanged). Due to the uneven distribution of data, logarithmic transformation was performed and the results are presented as geometric means (Xg) + − geometric standard deviation (SD). An unpaired, two-tailed Student’s t-test was applied; *p* < 0.05 was considered as statistically significant. RGE = relative gene expression

The expression of selected candidate genes was investigated by semi-quantitative (TaqMan) PCR using all available tissue samples (Fig. 7). Thirteen target genes were chosen that were predicted to be either upregulated or downregulated in particular contrasts based on the deep sequencing results. The functional groups chosen for validation of transcriptomics data included immune system, regulation of extracellular matrix and factors involved in steroid synthesis, including some transcriptional factors. Generally, a good correlation was found between transcriptomics and qPCR data. Thus, in the pairwise comparison “luteal regression over prepartum luteolysis”, upregulated expression of ECM2, GATA4, GATA6 and RXFP1 was found in samples derived from luteal regression, compared with natural prepartum luteolysis (for details, including statistical results, see Fig. 7a). On the other hand, the expression of immune and proinflammatory factors: MHCII, CXCL8 (IL8), IL1b, CCL3, CCL13 and SAA, was significantly higher during prepartum luteolysis (Fig. 7a).

When comparing the two groups “mid-gestation over luteolysis” (Fig. 7b), higher levels of JUN were detected in mid-pregnant dogs. The expression of IL1b, CCL3, MHCII and CCL13 was higher in samples collected during prepartum luteolysis. The expression of steroidogenic factor GATA4, which was predicted to be downregulated during prepartum luteolysis, did not differ between the two groups in qPCR.

As for the impact of antigestagen treatment on gene expression in mid-pregnant dogs (i.e., contrast “mid-gestation over antigestagen”; Fig. 7c), the expression of CCNA2, a factor involved in cell proliferation, was investigated and was more highly expressed (*P* < 0.0007) in the mid-pregnant group. Additionally, the expression of factors involved in steroidogenic and luteotropic actions, i.e., GATA4 and RXFP1, was examined and also found to be more highly represented in the mid-pregnant group. The same was found for the expression of proinflammatory factor CCL3, which was expressed more (*p* < 0.01) in samples from animals in which abortion was induced (for details see Fig. 7c).

For the last contrast “luteolysis over antigestagen”, higher expression of pro-inflammatory and acute phase reaction factors was confirmed during prepartum luteolysis (Fig. 7d). This concerns the expression of MHCII, CXCL8 (IL8), CCL3 and SAA. The expression of JUN was higher in the antigestagen-treated group. However, the mRNA levels of YY1 did not differ significantly between the two groups in qPCR.

## Discussion

Whereas in the dog the CL are the only major source of circulating P4 during pregnancy and in non-pregnant cycles, it is striking that the molecular and endocrine mechanisms regulating its life span are not yet fully understood. Because of similar P4 profiles these mechanisms must differ mostly during termination of luteal function. Consequently, here, for the first time a comparative, new generation sequencing analysis was performed to investigate the transcriptome of canine CL during the cessation of their function in pregnant and non-pregnant animals. Additionally samples from pregnant dogs in which prepartum luteolysis/abortion was induced at mid-gestation with the antigestagen aglepristone, were included.

One of our main goals was to compare samples collected during late luteal regression (day 65 after ovulation) with those from active prepartum luteolysis. At this point, it is important to emphasize that in the dog the prepartum luteolysis (characterized by actively decreasing P4 concentrations) already begins 12-42 h before any clinical and/or behavioral (e.g., nest building) signs of parturition become visible [36, 37]. Therefore, as presented here, it is extremely important to capture the right time point for collection of samples in order to validate them as being derived from prepartum luteolysis. Pairwise alignments were performed between the respective experimental groups.

When compared with prepartum luteolysis, the slow luteal regression in non-pregnant bitches was characterized by functional terms related to structural changes of the tissue, e.g., remodeling and organization of extracellular matrix. The steroid hormone-mediated signaling and activities, as well as factors related to regulation of gene expression, were also more highly represented in regressing CL. Genes involved in modification of matrix were represented, e.g., by PDGFRA (platelet-derived growth factor receptor α) or ECM2 (extracellular matrix protein 2). Interestingly, some of the nuclear steroidogenic receptors were detected within the overrepresented group of genes involved in structural changes in regressing canine CL, e.g., AR (androgen receptor), ESR1 (estrogen receptor 1, the gene encoding for estrogen receptor ERα) or NR3C1 (glucocorticoid receptor). In view of the postulated supportive effects of estrogens on luteal maintenance, based on circulating profiles of E2 closely matching those of P4 in pregnant and non-pregnant bitches, the diminishing effects of luteolysis on the ESR1 (ERα) expression drew our attention. The cAMP-mediated signaling was among the pathways more strongly represented during luteal regression than at prepartum luteolysis. The cAMP/PKA-related transcriptional activities are well known as the major positive regulators of steroidogenesis [38]. Thus, it can be concluded that the ongoing remodeling, morphogenesis and connective tissue development processes found in regressing canine CL are associated with higher functional and steroidogenic activities, compared with active prepartum luteolysis. This clearly can be seen in conjunction with the slowly ongoing functional and morphological changes associated with corpus albicans formation as a result of ageing of the steroidogenic apparatus and its replacement by matrix and connective tissue components.

The expression of selected target genes more highly represented in regressing canine CL was verified. Among these was ECM2, which has been proved to promote matrix assembly and cell adhesiveness [39]. We also examined the expression of RXFP1, a receptor for relaxin (RLN) proven to be involved in the maintenance of connective tissue extracellular matrix [40] and which influences the control of collagen turnover [41]. Importantly, in some species like pigs and rodents, a luteotropic function of intraluteally produced RLN has been implied [42, 43]. GATA4 and GATA6, both GATA binding proteins and transcriptional factors, are important for the synthesis of luteal P4 [44]. They are known to activate promoters of various steroidogenic genes, e.g., steroidogenic acute regulatory (STAR) protein [45], cholesterol side chain cleavage cytochrome P450 gene (CYP11A1) [46] or 3beta-hydroxysteroid dehydrogenase (3betaHSD) [47]. Another gene of possible functional importance with higher expression in the luteal regression group was SULT1E1 (sulfotransferase family 1E member 1 also known as estrogen sulfotransferase/EST), a key enzyme for catalyzing the sulfoconjugation of estrogens. This leads to their inactivation by preventing them from binding to their respective estrogen receptors, ERα/β [48]. The increased expression of SULT1E1 during luteal regression could thus be involved in the functional withdrawal of local estrogen effects in the canine CL. An additional role for this enzyme during the ongoing fatty degeneration of regressing/luteolytic canine CL appears plausible, as its involvement in positive regulation of adipogenesis has been shown, e.g., in humans [49]. This hypothesis, however, requires further verification.

A completely different picture regarding endocrine regulatory events emerges when functional terms and networks overrepresented during active prepartum luteolysis are considered. The vast majority of genes more highly represented in this group, compared with luteal regression, are related to immune and inflammatory response, including activation of leukocytes and acute-phase response. Also terms related to negative regulation of steroidogenesis, degeneration of mitochondria and apoptosis were found. The acute phase response is an immediate reaction based on inflammation due to various elicitors such as tissue injury, infection and trauma [50]. SAA (serum amyloid A protein) is one of the acute phase proteins. During inflammation, the baseline concentrations of this protein can increase by more than 1000-fold [51]. Here, the high abundance of the respective transcript was positively verified during active prepartum luteolysis in the dog by semi-quantitative PCR. Cytokines that are synthesized during an inflammatory process are the main stimulators of the acute-phase protein production, for example IL1β, IL6, TNFα, IFN-γ, TGFβ [52] and possibly IL8 [53]. The functional pathways and top upstream regulators detected by IPA software, as well as overrepresented networks detected by Cytoscape during luteolysis, indicate induction of the luteal immune system at prepartum. Thus, besides the already mentioned activation of IL6 and IL8 signaling, in addition the NFκB signaling pathway and overrepresentation of the TNF and LPS systems were found, as well as the induction of TGFβ pathway. Interestingly, the TGFβ signaling was shown to possess profibrotic and anti-angiogenic potential, contributing thereby to structural and functional luteolysis in the bovine CL [54, 55]. Among the selected genes investigated in our study by semi-quantitative PCR and belonging to the immune response were: CXCL8 (chemokine (C-X-C motif) ligand 8; IL-8), IL1B (interleukin 1 beta), MHCII (major histocompatibility complex, class II), CCL3 (chemokine (C-C motif) ligand 3) or CCL13 (chemokine (C-C motif) ligand 13). The proinflammatory cytokine IL-8 is synthesized predominantly by macrophages and neutrophils, which are recruited to the site of inflammation [56]. It is known for its chemotactic activity directed towards T cells [57]. CCL3 and CCL13 are both chemokine ligands, whose main task is to act as chemoattractants to draw active immune cells to the site of inflammation. MHCII proteins, in turn, are located on antigen-presenting cells and play important roles during initiation of the immune response [58].

Cumulatively, it becomes apparent that active prepartum luteolysis in the dog represents a highly inflammatory, acute event, involving activation of multiple pathways responsible for the immune response. This reaction seems to be triggered by concomitantly increasing utero-placental PGF2alpha. It is noteworthy that, regarding potential involvement of the immune system in the regulation of luteal function during active prepartum luteolysis in the dog, our results corroborate observations made previously in other species, e.g. in rodents, humans or farm animals like sheep, pigs, cattle and horses, as reviewed in [59].

The acute inflammatory nature of luteolysis also becomes obvious when comparing the mid-gestation group with prepartum luteolysis. Whereas in mid-pregnant dogs the remodelling processes associated with structural CL regression were already clearly visible, also in this contrast the prepartum luteolysis was characterized by inflammatory- and acute-phase responses, including NFκB-, TNF- and LPS- mediated effects, as well as by apoptotic signaling. Similarly, the expression of selected target genes (IL1b, CCL3, CCL13 and MHCII) was confirmed, displaying higher levels during natural luteolysis. TGFβ signaling was represented both by genes relating to structural remodelling during mid-gestation (detected by Bioconductor), and to inflammatory pathways found by IPA software during prepartum luteolysis. This further underlines its possible involvement in morphological and functional cessation of the canine CL life span.

Antigestagen treatment results in preterm luteolysis/abortion [10, 25] and accelerates luteal regression in mid-luteal, non-pregnant bitches [60]. The underlying molecular and endocrine mechanisms associated with the functional withdrawal of P4 remain, however, not completely understood. This prompted us to investigate the impact of aglepristone on the luteal transcriptome in mid-pregnant dogs, comparing them with non-treated controls. Therefore, the experimental contrast “mid-gestation over antigestagen” was established. However, this contrast resulted in the highest variability of gene expression. Thus, despite the relatively high number of genes, the expression of which differed at the applied level of *p* < 0.01 (429 genes), when the corrected *p*-value (FDR) of <0.1 was applied, the list of DEG became restricted to 92 genes. This strongly limited interpretation of these results. It appears, however, that application of antigestagen to mid-pregnant dogs results in induction of the immune response and suppresses terms related to proliferation and protein metabolism. These effects were mirrored by higher expression of selected target genes validated by semi-quantitative PCR. Thus, the cyclin A2 (CCNA2), an important positive regulator of cell cycle progression, was more highly expressed in the CL of control mid-pregnant dogs. Interestingly, however, although it was significantly enriched at the level of *p* < 0.01, expression of this gene did not pass the initially applied FDR <0.1 selection criterion. Additionally, we found increased expression of transcriptionally and steroidogenically active GATA4 and RXFP1, during mid-pregnancy. On the other hand, the immune system-derived CCL3 was elevated in treated CL. Cumulatively, based on the results presented herein, and despite the high variability of results obtained in the discussed contrast (i.e., mid-gestation over antigestagen), the luteotropic function of P4 within canine CL may be related to positive regulation of the cell cycle, proliferation and transcriptional activity, and anti-inflammatory effects.

Interestingly, as revealed in the next comparison, although it led to suppression of luteal steroidogenesis, when compared with the natural prepartum luteolysis, the antigestagen treatment evoked weaker immune and inflammatory reactions. Instead, as in the previous contrast, the inhibitory effects related predominantly to cell proliferation, also inhibition of transcription and gene expression was prevalent. Additionally, Cytoscape assigned genes associated with functional networks relating to the response to TGFβ in the antigestagen-treated group. Among these were genes associated with structural remodelling of tissue such as ADAMTSL2. Among representative genes expressed more during natural luteolysis and positively validated by semi-quantitative PCR were immunoreactive factors involved in the acute response, such as CXCL8 (IL8) and SAA. Besides the acute inhibition of P4 production during natural luteolysis, the functional withdrawal of P4 during this time was marked by higher expression of transcripts encoding for CYP17A1 (steroid-17*alpha*-hydroxylase) enzyme. Acting upon P4, CYP17A1 catalyzes its conversion to the less biologically potent 17-OHP (17-hydroxyprogesterone).

Changes induced in luteal transcriptomes, commonly regulated during normal and induced luteolysis, were compared using Venn diagrams. First, we aimed to compare the changes induced by endogenous PGF2alpha in late regressing CL (i.e., during natural luteolysis), with the antigestagen-mediated changes evoked in fully active CL. Therefore, the two contrasts “luteal regression over luteolysis” and “mid-gestation over antigestagen” were chosen. We found two genes, CYP17A1 and SULT1E1, which were commonly represented more during luteal regression and mid-gestation over their luteolytic counterparts. Thus, both genes that were less expressed in CL dominated by apoptosis are involved in controlling luteal steroidogenic capability. Genes which were commonly more highly expressed in luteolytic groups included some of the immune system-relevant factors like C5AR2 (CD88).

In the next comparison we were interested to find the genes characteristic of antigestagen-mediated effects. The two contrasts submitted to the analysis were: “luteolysis over antigestagen” and “mid-gestation over antigestagen”. We found CYP17A, being commonly less expressed in antigestagen-treated samples in both contrasts. Among genes commonly more represented in the antigestagen groups of this contrast were CSRNP3 (known as TGF-β-induced apoptosis protein 2) and ELL2 (elongation factor for RNA polymerase II 2), a regulator of gene transcription.

## Conclusions

**Fig. 8:**
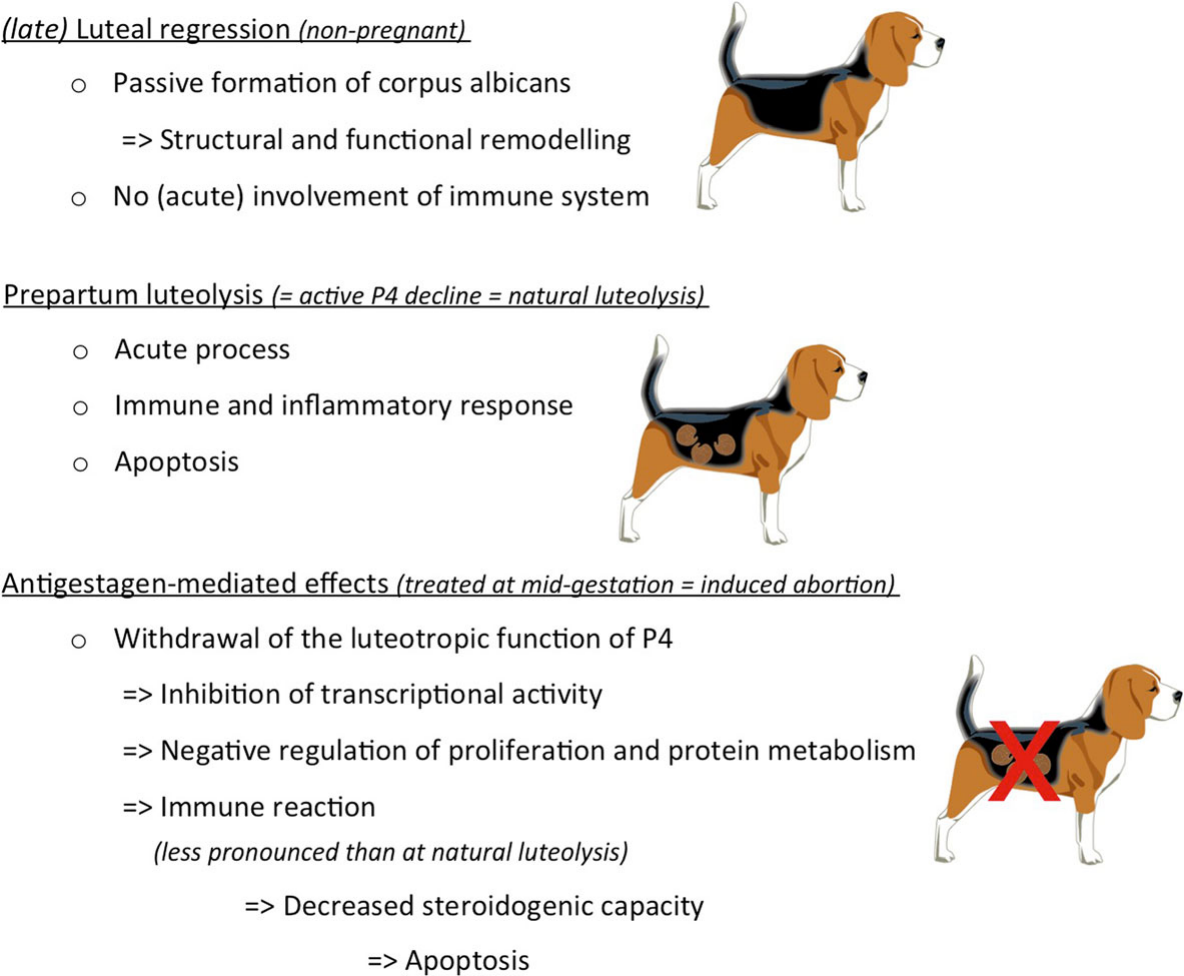
A cumulative presentation of major conclusions drawn from the present study is shown. Thus, luteal regression in non-pregnant bitches appears to be a slowly ongoing, passive degenerative process leading to corpus albicans formation, associated with structural remodelling processes in the absence of an acute inflammatory reaction. The latter, i.e., acute inflammation, is observed during prepartum luteolysis, and is most probably caused by PGF2alpha of utero-placental origin. The antigestagen-mediated effects seem to relate primarily to inhibition of the luteotropic function of progesterone (P4), rather than to the inflammatory reaction evoked by its withdrawal

Herein, deeper insights have been obtained into possible cellular mechanisms governing the luteal life span in the domestic dog during pregnancy and in non-pregnant cycles. The most important conclusions from our study are summarized in Fig. 8.

Clearly, the data obtained by RNA-Seq are not definitive, as they need to be further substantiated by studies involving larger numbers of animals and by presenting more mechanistic approaches, including in vivo and in vitro studies.

Nevertheless, however, the analysis of transcriptomes presented herein supports our previously postulated hypothesis describing luteal regression in non-pregnant dogs as a degenerative process devoid of the acute luteolytic principle and without acute involvement of the immune system as observed prepartum [1]. The contribution of the immune system seems to be critical in the PGF2alpha-mediated active prepartum luteolysis, which appears to be an acute immune process. The antigestagen-mediated effects may primarily result in the inhibition of gene expression and cell proliferation, as well as the inflammatory response. These effects cumulatively point towards the withdrawal of the luteotropic function of P4.

The P4-mediated effects can be especially important for understanding some of the clinical conditions associated with the negative pregnancy outcomes, such as luteal insufficiency which affects some bitches.

Finally, several differentially expressed genes were identified within the experimental groups representing different functional pathways and networks and being possibly involved in the maintenance and cessation of canine luteal function. Some of them deserve closer attention for determining future research directions, like the TGFβ-mediated pathways. Similarly, the cellular origin and functional interplay between different immune system-derived factors need further clarification. Future studies will also need to focus on translating information obtained from investigating the transcriptome to functional studies at the protein level.

## Additional files

Additional file 1: List of DEG found in the contrast “luteal regression over luteolysis”. All genes detected with *p* < 0.01 passed the FDR < 0.1 selection (adjusted *p*-values). (XLSX 141 kb)

Additional file 2: List of DEG found in the contrast “mid-gestation over luteolysis”. All genes detected with *p* < 0.01 passed the FDR < 0.1 selection (adjusted *p*-values). (XLSX 154 kb)

Additional file 3: List of DEG found in the contrast “mid-gestation over antigestagen”. Shown are the following lists: (1–2) list of genes with *p* < 0.01; (3–4) list of genes with adjusted *p*-values (i.e. FDR <0.1). (XLSX 71 kb)

Additional file 4: List of DEG found in the contrast “luteolysis over antigestagen”. All genes detected with *p* < 0.01 passed the FDR < 0.1 selection (adjusted *p*-values). (XLSX 133 kb)

Additional file 5: Shown are the following lists: (1) representative genes involved in particular GO terms; (2) representative genes involved in particular networks; (3) representative pathways, genes and top upstream regulators. (XLSX 21 kb)

Additional file 6: Venn-diagrams, combined results for Figs. 3 and 4. An overview of overlapping genes commonly expressed between the selected contrasts: (contrast 1) “luteal regression over luteolysis” and (contrast 2) “mid-gestation over antigestagen”, or (contrast 1) “luteolysis over antigestagen” and (contrast 2) “mid-gestation over luteolysis”. There are separate lists for each set threshold: genes expressed solely in either of the contrasts, and genes commonly expressed in both contrasts. (XLSX 67 kb)
